# Comparison of reconstruction nails versus dual implants in the treatment of ipsilateral femoral neck and shaft fractures in adults: a meta-analysis and systematic review

**DOI:** 10.1186/s12891-023-06933-6

**Published:** 2023-10-09

**Authors:** Yongchao Zhao, Jian Li, Yadong Liu, Guanlu Cui, Zhengwei Li

**Affiliations:** 1 Department of Orthopedics, The Second Hospital of Jilin University, Changchun, Jilin Province China; 2 Department of Emergency and Critical Care, The Second Hospital of Jilin University, Changchun, China

**Keywords:** Ipsilateral, Femoral neck fractures, Femoral shaft fractures, Reconstruction nails, Dual implants

## Abstract

**Objective:**

There is no consensus on the optimal treatment for ipsilateral femoral neck and shaft fractures. This meta-analysis aims to assess the effectiveness of reconstruction nails and dual implants in treating ipsilateral femoral neck and shaft fractures to provide a basis for decision-making when selecting the optimal approach.

**Methods:**

Relevant articles were retrieved from Pubmed, Embase, and Cochrane databases using the keywords "neck of femur", "shaft" and "fracture fixation" from inception until November 17, 2022. The screening process of the studies was conducted independently by two assessors, who assessed each study's eligibility and two assessors assessed the quality. Then compared differences in outcome measures using RevMan 5.3 software.

**Results:**

A total of ten retrospective cohort studies were included. There were no significant differences in union time, union rate, union-related complications (malunion, nonunion, delayed union) of femoral neck and shaft fractures, osteonecrosis of the femoral head, and functional outcomes (Friedman-Wyman scoring system) (*P* > 0.05).

**Conclusion:**

Our pooled estimates indicated that reconstruction nails and dual implants for ipsilateral femoral neck and shaft fractures could yield satisfactory surgical results, and that there is no difference between the two treatment methods.

**Trial registration:**

This meta-analysis was registered on the PROSPERO website (registration number: CRD42022379606).

**Supplementary Information:**

The online version contains supplementary material available at 10.1186/s12891-023-06933-6.

## Introduction

Fractures involving the ipsilateral femoral neck and shaft are uncommon and comprise approximately 1% to 9% of all femur fractures [1–3]. These injuries typically result from high-energy trauma and are more common in young adult males who also sustain head, thoracic, abdominal and knee injuries [3–9], resulting in a considerable financial burden it on patients, families and health system.

The goal of the treatment is the excellent reduction and stable fixation of the femoral neck and shaft fractures, allowing patients to engage in functional exercises at an early stage [5–8, 10]. Given that surgical management of ipsilateral femoral neck and shaft fractures yields significantly better results than non-operative treatment [11–14], surgical treatment is indicated as soon as the patient's general condition permits [15]. Casey et al. [16] reported that life-threatening pulmonary complications developed in nine patients treated non-operatively after ipsilateral femoral neck and shaft fractures. Early surgical intervention offers several benefits, such as easier nursing care, early mobilization and rehabilitation of patients, reducing morbidity and mortality from complications, and ultimately improving overall outcomes [13, 17]. However, the treatment of combined neck and shaft fractures has been controversial because there is no consensus regarding its standardized treatment [18], including the optimal implant to be used, the optimal timing of surgery, and which fracture to be stabilized first [15, 19]. This meta-analysis focuses on the optimal implant to be used. It is widely acknowledged that there are two types of treatment, i.e. single versus double fixation. Single fixation is a reconstruction nail to fix both the femoral neck and shaft fracture simultaneously. Double fixation using two devices, i.e. the femoral shaft fracture is fixed with retrograde or anterograde intramedullary nail or plate, and the femoral neck fracture is fixed with cancellous screws or dynamic/sliding hip screws (DHS/SHS) [2, 5, 9, 10, 20–22].

Bose et al. [23] and Leung et al. [24] recommended using reconstruction nails for ipsilateral femoral neck and shaft fractures. They argued that reconstruction nails could facilitate closed reduction and stable fixation of both fractures and help control the femoral shaft's angulation, shortening, and rotation. In addition, this fixation method required less incision, less blood loss, and less infection and could be used for the biological fixation of fractures [5, 15, 25, 26]. However, the surgical procedure of reconstruction nails is complicated, the learning curve is long, and the technical requirements for surgeons are high, increasing susceptibility to complications such as femoral head osteonecrosis and bone nonunion [1, 27, 28]. Anterograde intramedullary nails combined with cancellous screws can effectively treat femoral shaft fractures, while fixation of the femoral neck using this approach results in instability [12, 29, 30]. Retrograde intramedullary nails are considered reliable for fixing shaft fractures without affecting the selection of proximal implants [10]. However, this approach may also increase the risk of postoperative knee complications [31]. Ricci et al. [31] found that the incidence of knee pain after retrograde intramedullary nails was 36%. Plate fixation has long been used for femoral shaft fracture fixation with separate fixation of the femoral neck [1, 20], especially in cases of open fracture requiring wide exposure for fracture debridement [2].This operation is simple, easy, reliable for fracture fixation, and can prevent shortening effectively [19, 32]. Despite its potential benefits, plate fixation has several drawbacks, such as excessive soft tissue separation, compromised blood supply at the fracture site, and eccentric fixation, which increase the risk of infection and nonunion [2, 26, 29, 33]. Therefore, the optimal fixation strategy for ipsilateral femoral neck and shaft fractures remains controversial [2, 5, 10, 20–22]. This meta-analysis aims to analyze the prognosis and complications of reconstruction nails and double implants in treating these fractures.

## Materials and methods

This meta-analysis was conducted in the Preferred Reporting Items for Systematic Reviews and Meta-Analyses (PRISMA) statement [34]. Since it is a secondary literature analysis, no ethical review was required.

### Inclusion criteria and exclusion criteria

Inclusion criteria: Randomized controlled trials (RCTs) or observational studies; Ipsilateral femoral neck and shaft fractures in adults; The experimental group was treated with reconstruction nails, and the control group was treated with dual implants; At least one outcome measure (fracture union time, fracture union, fracture malunion or nonunion or delayed union, osteonecrosis of the femoral head) was reported; The language of the article is English or Chinese. Exclusion criteria: Patients with a pathological fracture; Articles without full-text; Valid data could not be extracted from the study for this meta-analysis.

### Search strategy

We used the keywords "neck of femur", " shaft " and "fracture fixation" to search articles in Pubmed, Embase, and Cochrane databases from inception until November 17, 2022. The references of relevant reviews and systematic reviews were manually searched. The detailed search strategies are included in the additional file 1.

### Selection process

Two assessors (Yongchao Zhao and Jian Li) independently screened the literature according to the inclusion/exclusion criteria, and a third person (Zhengwei Li) dealt with the controversial literature.

### Data extraction

Data from the included literature was independently extracted by two assessors (Yongchao Zhao and Jian Li) and recorded in Microsoft Excel files. Data extracted included: first author, year of publication, participant characteristics (age, sex, mechanism of injury), intervention (reconstructive nails and double implants), average operation time, outcome data (intraoperative blood loss, fracture healing, fracture average union time, osteonecrosis of the femoral head, postoperative complications), and functional outcome (Friedman-Wyman score). Points of disagreement were reconciled by a discussion with a third investigator (Zhengwei Li). If the data reported in the article were incomplete, the corresponding author was contacted by e-mail to obtain the original data; if no response was received from the authors, the method described in the Cochrane Handbook for Systematic Reviews of Interventions was used to convert the data, and the standard deviation (SD) was estimated based on the confidence interval (CI).

### Outcome measures

The following five indexes were used to analyze and compare the efficacy of internal fixation between the two groups.

### Fracture union and fracture union time

Fracture union can be defined as the restoration of mechanical properties of the bone, such as strength and stiffness [35], and osseous bridging of three or four cortices on anteroposterior and lateral radiographs [36]. All patients in this study received surgical treatment. Successful healing of fractures in this study was defined as an osseous union within six months after the index surgery [36]. Delayed union of a long bone is considered when the fracture has not fully united after six months [37], while nonunion is defined as no indication of bone healing until nine months after surgery [8]. Fracture union in the studies included in this meta-analysis also included patients with malunion and delayed union. These can indicate the quality of surgery and the speed of fracture healing.

### Fracture union complications

Fracture union complications include malunion, delayed union, and nonunion. Delayed union and nonunion have been described above. Malunion is the fracture heals in an abnormal position, resulting in a combination of angulation, rotation, and length discrepancies [38], and imaging studies may show deformities [39]. Complications are essential indicators for evaluating the safety of surgery.

### Osteonecrosis of the femoral head

The common clinical manifestations of femoral head osteonecrosis are deep pain in the groin and limited hip motion, while radiographic findings include cystic and sclerotic changes in the femoral head [40]. It has been established that the incidence of osteonecrosis of the femoral head is associated with the quality of reduction [16, 22]. Thus, a high incidence of osteonecrosis indicates poor reduction quality, which can be used to assess the reduction effect.

### Friedman-Wyman scoring system

The Friedman-Wyman scoring system assesses functional outcomes in three aspects: activities of daily living (ADL), pain, and hip or knee motion. The results are categorized as good, fair, or poor [41]. To facilitate subsequent analysis, the hierarchical data was subjected to binary processing. In this study, "good" was defined as no limitations in ADL, no pain, and a maximum 20% loss of hip or knee motion, with the rest considered "poor". The Friedman-Wyman scoring system is indicative of the extent of functional recovery of patients after surgery.

### Quality assessment

Two evaluators (Yadong Liu and Guanlu Cui) independently evaluated the quality of selection, comparability, and exposure of the ten cohort studies included in the meta-analysis using the Newcastle–Ottawa scale (NOS) [42]. Disagreements regarding eligibility were resolved by discussion with a third researcher (Zhengwei Li).

### Statistical analysis

Standardized mean difference (SMD) and 95%CI were used as combined effect indicators for continuous variables; Risk ratio (RR) and 95%CI were used for dichotomous variables. Review Manager (Revman) Version 5.3 (The Nordic Cochrane Centre, The Cochrane Collaboration) was used for the meta-analysis. Dichotomous variables were analyzed by the Mantel–Haenszel method, and the results were described by RR and 95%CI. Continuous variables were analyzed by inverse variance, and the results were described by SMD and 95%CI. The chi-square test was adopted to assess heterogeneity, with a threshold of *P* < 0.05. A value of I^2^ > 50% indicated high heterogeneity. A random effects model was utilized when the I^2^ value was greater than 50%; otherwise, a fixed effects model was employed. The results of each variable were visualized in a forest plot. Sensitivity analysis was conducted for outcome indicators with significant heterogeneity, and the included studies were excluded one at a time to determine the source of heterogeneity. Funnel plots were utilized to test for bias in the included studies.

## Results

### Study selection

**Fig. 1:**
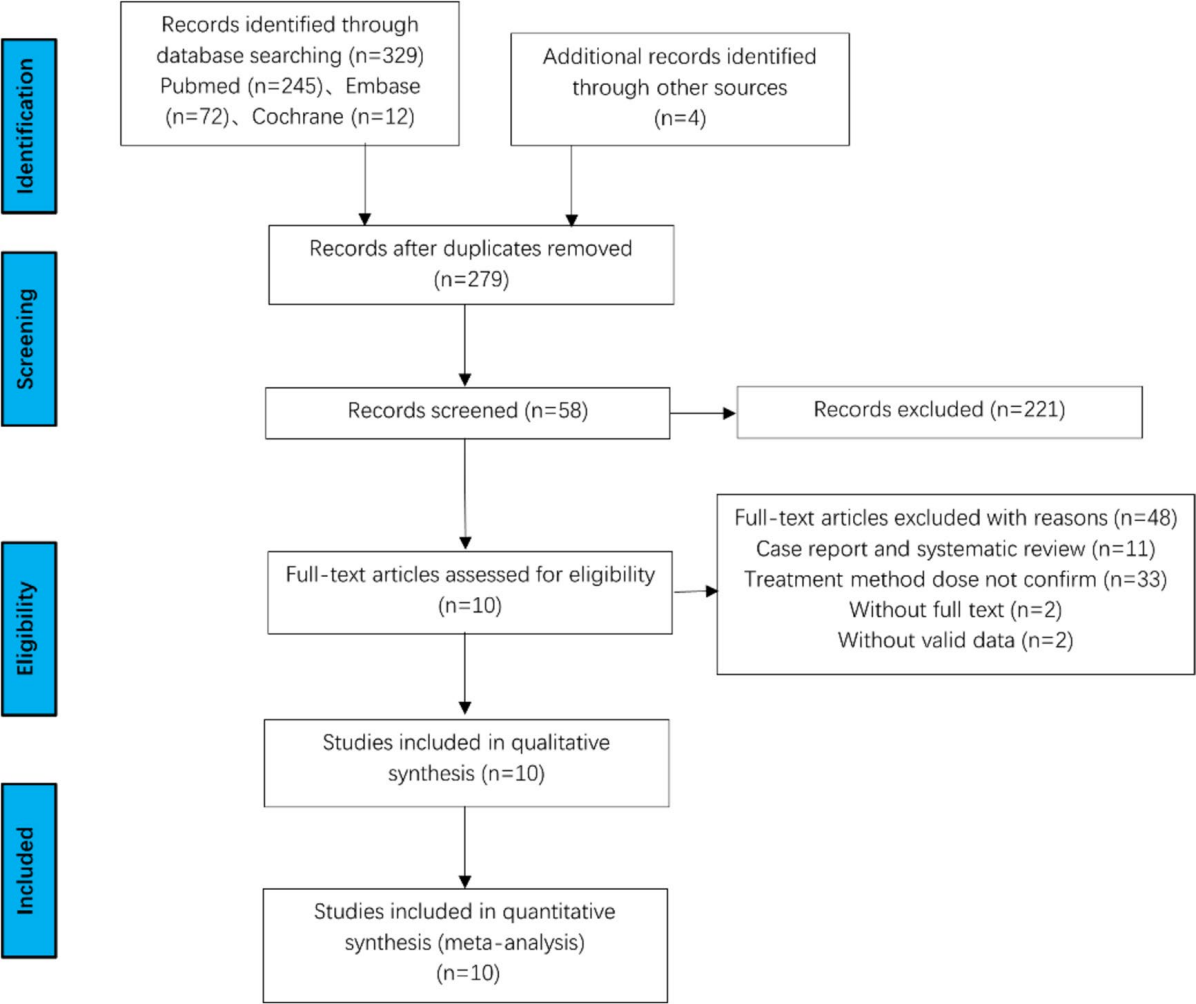
Flowchart of literature retrieval and selection

The literature search yielded a total of 333 studies. After excluding duplicates, 279 studies were retained. After reading titles and abstracts, 58 studies remained. We next excluded 11 case reports or systematic reviews, 33 studies with the reason that their treatment methods did not confirm, two studies without full text and two studies without valid data (Fig. 1).

### Study characteristics

The ten included studies [5–8, 10, 14, 18, 19, 43, 44] published between 2008 and 2022 comprised 404 participants. Detailed information on sample size, sex, mechanism of injury, interventions, and follow-up time of the ten studies are provided in Table 1.

**Table 1:** Characteristics of the included studies

Trails	Cases	Sex	Age (Year)	Mechanism	Interventions		Follow-up Time (M)
	T/C	M/F	T/C		T	C	T/C
Liu (2008) [43]	8/16	—	—	①②	RC	ANC、PC	—
Singh (2008) [5]	12/15	24/3	37.9/33.2	②	RC	PC、DHSP	27.1/24.2
Bedi (2009) [10]	9/28	18/19	18	—	RC	RNC、SHSR	—
Cannada(2009) [6]	16/72	—	36	①②	RC	ANC、RNC、SHSR、SHSN	17
Tsai (2009) [7]	5/38	28/15	43	①②	RC	DHSP、PC、ANC	48
Kharel (2017) [19]	11/13	13/11	34/31	②	RC	PC、DHSP	20.2/19.9
Mohapatra (2017) [18]	10/8	14/4	31.2/32	①	RC	PC、DHSP	28/23.4
Feifan (2020) [44]	11/22	24/9	40.1/45.2	①②③	RC	PC、RNC	—
Alborno (2022) [14]	24/12	30/6	38.1/36.3	①②	RC	ANC、RNC、SHSR、PC	7.3
Oh (2022) [8]	14/60	60/14	43.6	①②	RC	PC、RNC、DHSR、DHSP	33

*ANC* Antegrade nailing + cancellous screws, *C* Control group, *DHSP* Dynamic hip screws + plate, *DHSR* Dynamic hip screws + retrograde femoral nailing, *M/F* Male/female, *PC* Plate + cancellous screws, *RC* Reconstruction nails, *RNC* Retrograde nailing + cancellous screws, *SHSR* Sliding hip screws + retrograde intramedullary nail, *SHSN* Sliding hip screws + flexible nails, *T* Treatment group, *T/C* Treatment group /control group

①: falling from a height; ②: high-energy trauma in road traffic accidents; ③: heavy objects smashed

### Quality assessment

The quality of the included ten cohort studies was assessed using the NOS [42]. A score ranging from five to nine was categorized as high quality. Following the NOS analysis, all ten studies were considered of high quality. Detailed results are presented in Table 2.

**Table 2:** Quality assessment of the included studies

Studies	Selection	Comparability	Outcome	Total
Liu (2008) [43]	3	1	2	6
Singh (2008) [5]	2	1	3	6
Bedi (2009) [10]	4	1	1	6
Cannada (2009) [6]	4	1	2	7
Tsai (2009) [7]	3	1	3	7
Kharel (2017) [19]	2	1	3	6
Mohapatra (2017) [18]	2	2	2	6
Feifan (2020) [44]	3	2	2	7
Alborno (2022) [14]	4	1	3	8
Oh (2022) [8]	3	1	3	7

## Results of studies

### Fracture union

**Fig. 2:**
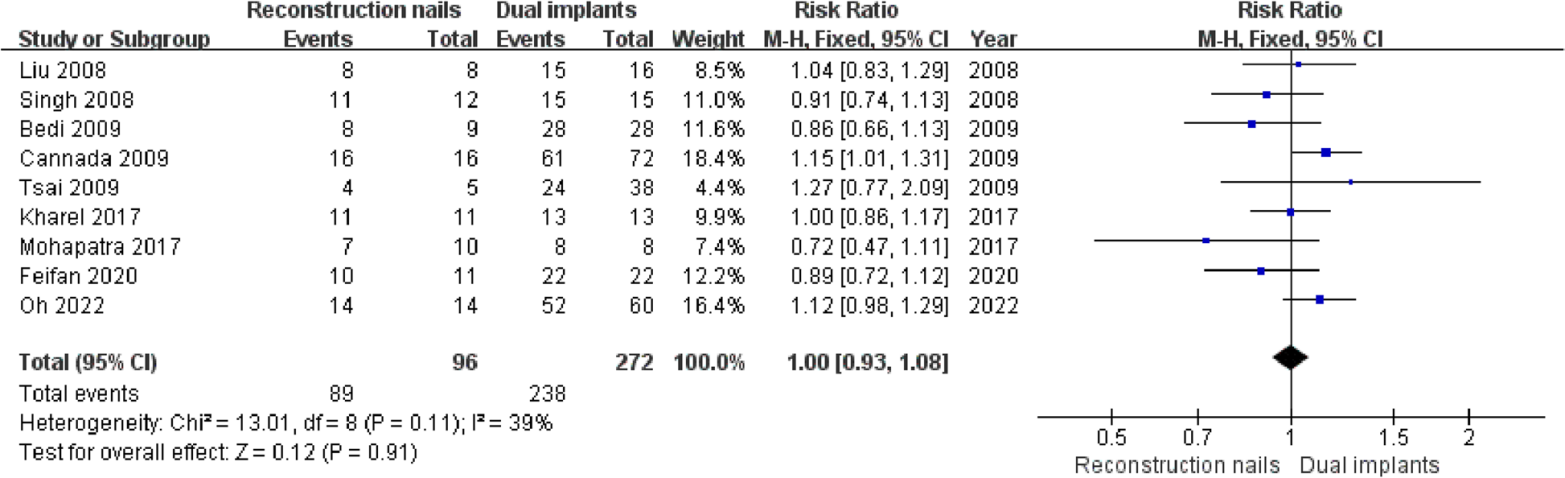
Forest plot for femoral neck fracture union

**Fig. 3:**
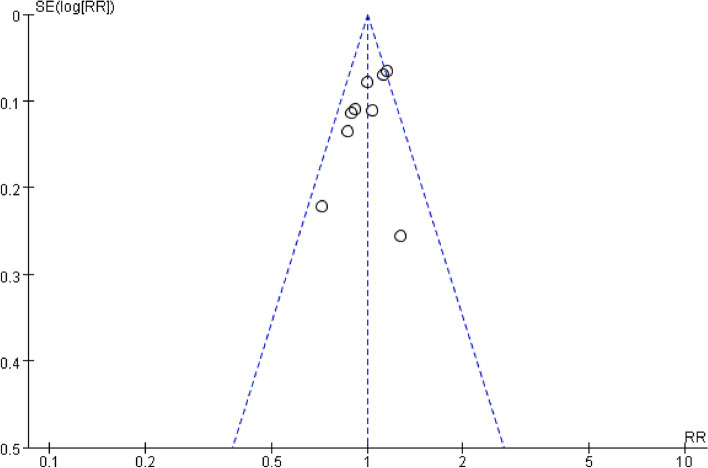
Funnel chart for femoral neck fracture union

Nine studies [5–8, 10, 18, 19, 43, 44] reported femoral neck fracture union, including 96 cases in the reconstruction nails group and 272 cases in the dual implants group. The studies demonstrated good homogeneity (I^2^ = 39%, *P* = 0.11). Thus, a fixed effects model was employed. As shown in Fig. 2, the pooled estimates results revealed no significant differences in femoral shaft fracture union rates (*P* > 0.05) between the two groups (RR = 1.00, 95%CI [0.93, 1.08], *P* = 0.91). A symmetrical funnel plot (Fig. 3) was generated, indicating that publication bias was not significant.

**Fig. 4:**
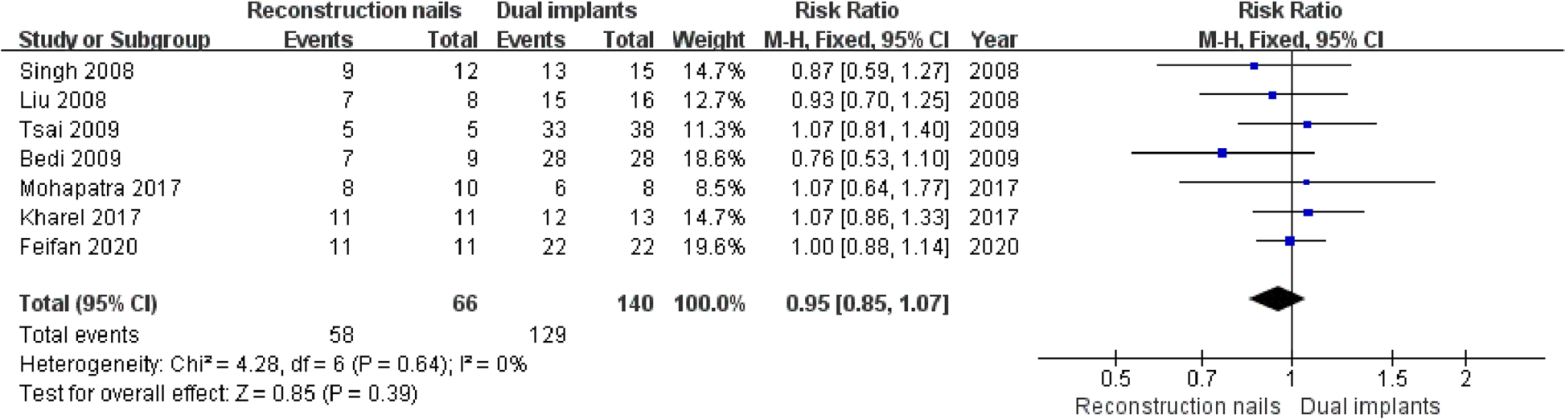
Forest plot for femoral shaft fracture union

Seven studies [5, 7, 10, 18, 19, 43, 44] reported femoral shaft fracture union, including 66 cases in the reconstruction nails group and 140 cases in the dual implants group. The studies demonstrated good homogeneity (I^2^ = 0%, *P* = 0.64). Thus, a fixed effects model was employed. As shown in Fig. 4, the pooled estimates results revealed no significant differences in femoral shaft fracture union rates (*P* > 0.05) between the two groups (RR = 0.95, 95%CI [0.85, 1.07] and *P* = 0.39).

### Osteonecrosis of the femoral head

**Fig. 5:**
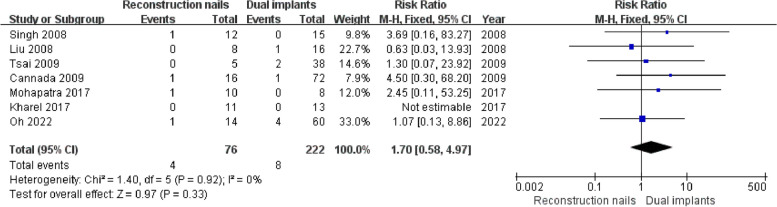
Forest plot for osteonecrosis of the femoral head

Seven studies [5–8, 18, 19, 43] reported osteonecrosis of the femoral head, including 76 cases in the reconstruction nails group and 222 cases in the dual implants group. Since there was good homogeneity among all studies (I^2^ = 0%, *P* = 0.92), a fixed effects model was selected. The pooled results revealed no significant differences (*P* > 0.05) in femoral head osteonecrosis between the two groups (RR = 1.70, 95%CI [0.58, 4.97], *P* = 0.33) (Fig. 5).

### Fracture union-related complications

**Fig. 6:**
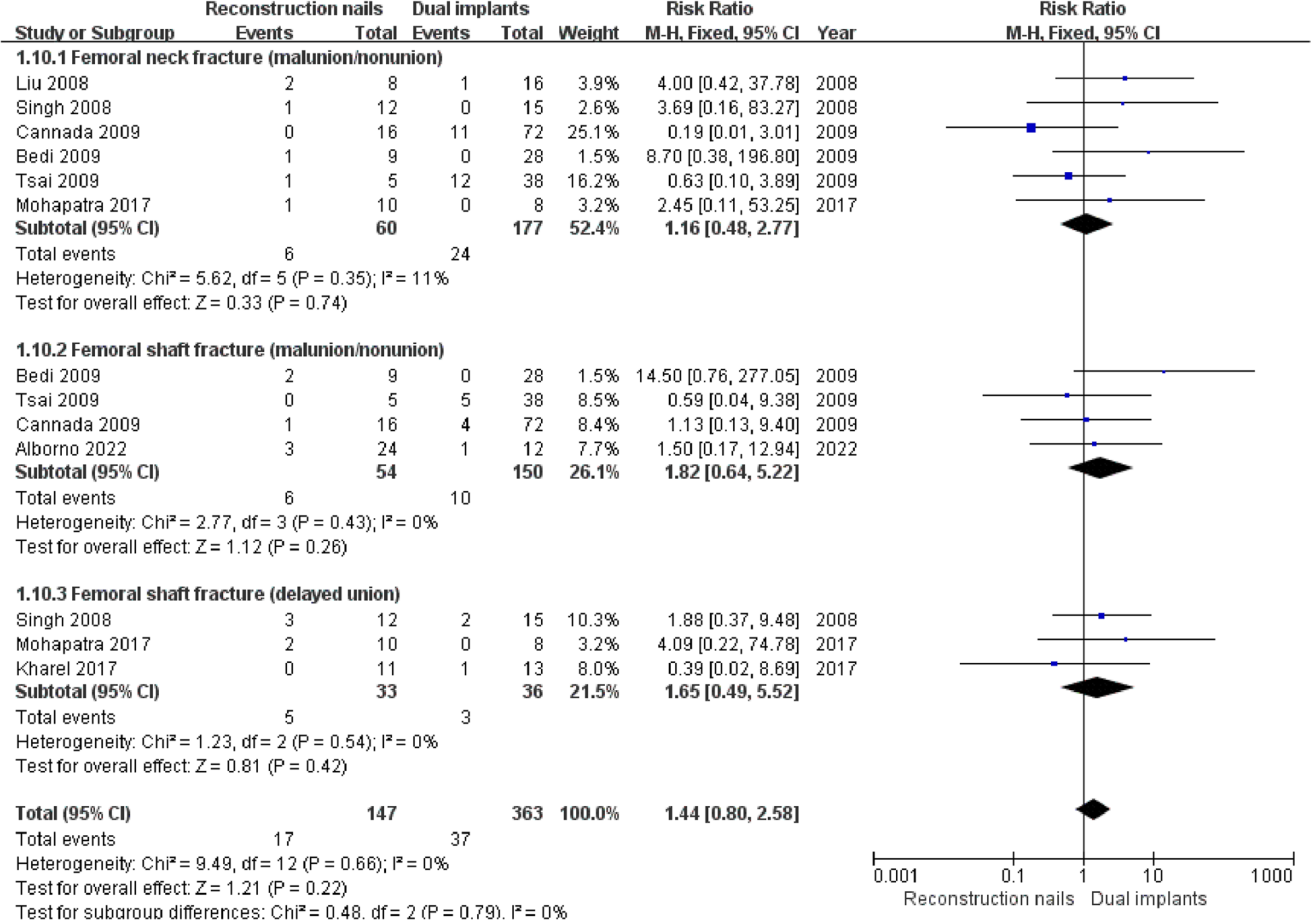
Forest plot for Fracture union-related complications

Six studies [5–7, 10, 18, 43] reported femoral neck fracture malunion, nonunion, including 60 cases in the reconstruction nails group and 177 cases in the dual implants group. No significant heterogeneity was observed among all studies (I^2^ = 11%, *P* = 0.35), and the fixed effects model was applied. The meta-analysis revealed no significant differences (*P* > 0.05) in femoral neck fracture malunion, nonunion rates between the two groups (RR = 1.16, 95%CI [0.48, 2.77], *P* = 0.74) (Fig. 6).

Four studies [6, 7, 10, 14] reported femoral shaft fracture malunion, nonunion, including 54 cases in the reconstruction nails group and 150 cases in the dual implants group. The fixed effects model was applied when there was no significant heterogeneity among these studies (I^2^ = 0%, *P* = 0.43). As shown in Fig. 6, no significant differences (*P* > 0.05) in femoral shaft fracture malunion, nonunion rates were observed between the two groups (RR = 1.82, 95%CI [0.64, 5.22], and *P* = 0.26).

Three studies [5, 18, 19] reported femoral shaft fracture delayed union, including 33 cases in the reconstruction nails group and 36 cases in the dual implants group. Good homogeneity was observed among all studies (I^2^ = 0%, *P* = 0.54); thus, the fixed effects model was selected. As shown in Fig. 6, our pooled estimates revealed no significant differences (*P* > 0.05) in femoral neck fracture delayed union rates (RR = 1.65, 95%CI [0.49, 5.52], and *P* = 0.42) between the two groups.

### Fracture union time

**Fig. 7:**
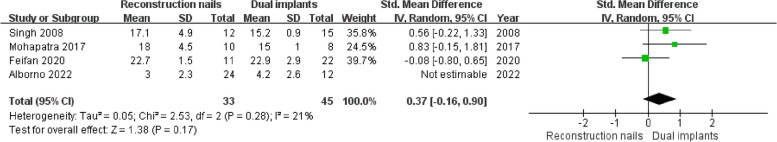
Forest plot for femoral neck fracture union time

Four studies [5, 14, 18, 44] reported femoral neck fracture union time, including 57 cases in the reconstruction nails group and 57 cases in the dual implants group. Heterogeneity was observed among the studies (I^2^ = 53%, *P* = 0.09). Thus the random effects model was selected. There was no significant difference between the reconstruction nails group and the dual implants group (SMD = 0.15, 95%CI [-0.42, 0.72], *P* = 0.61). To find the source of heterogeneity, sensitivity analysis was conducted. After the cohort study by Alborno (2022) was removed, the I^2^ was reduced to 21%, showing good homogeneity (*P* = 0.28), and there was no significant difference in femoral neck fracture union time between the reconstruction nails group and the dual implants group (SMD = 0.37, 95%CI [-0.16, 0.90], *P* = 0.17) (Fig. 7).

**Fig. 8:**
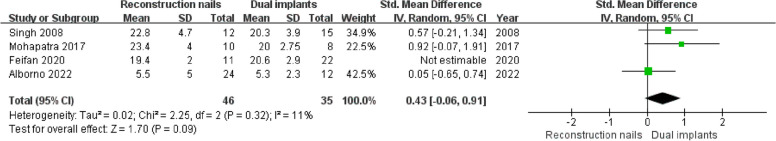
Forest plot for femoral shaft fracture union time

Four studies [5, 14, 18, 44] reported femoral shaft fracture union time, including 57 cases in the reconstruction nails group and 57 cases in the dual implants group. The random effects model was selected since heterogeneity was observed among the studies (I^2^ = 50%, *P* = 0.11). There was no significant difference between the reconstruction nails group and the dual implants group (SMD = 0.22, 95%CI [-0.34, 0.78], *P* = 0.45). To find the source of heterogeneity, sensitivity analysis was conducted. After the cohort study in which Feifan (2020) was removed, the I^2^ was reduced to 11%, showing good homogeneity (I^2^ = 11%, *P* = 0.32), and there also was no significant difference in femoral shaft fracture union time between the reconstruction nails group and the dual implants group (SMD = 0.43, 95%CI [-0.06, 0.91], *P* = 0.09) (Fig. 8).

### Functional outcomes (Friedman-Wyman scoring system)

**Fig. 9:**
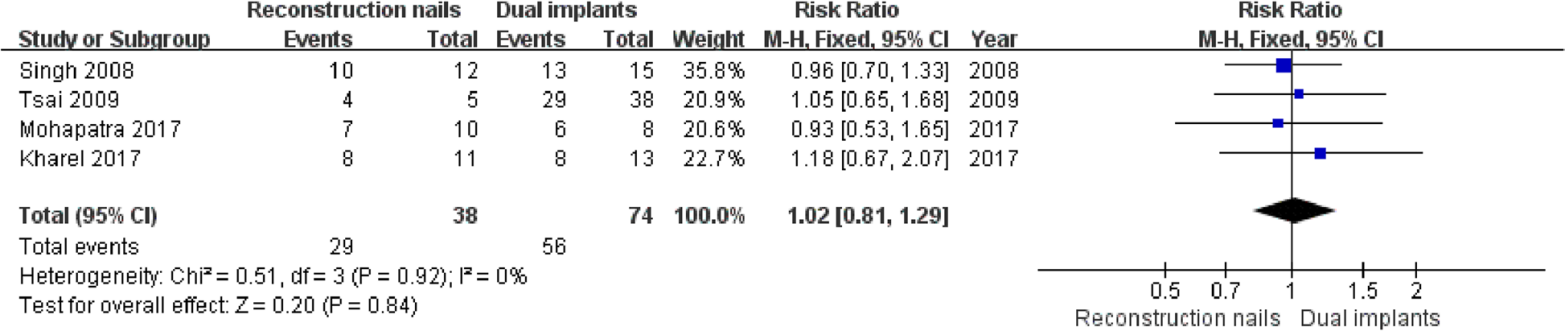
Forest plot for functional outcomes

Four studies [5, 7, 18, 19] reported femoral shaft fracture delayed union, including 38 cases in the reconstruction nails group and 74 cases in the dual implants group. Given that there was good homogeneity among all studies (I^2^ = 0%, *P* = 0.92), a fixed effects model was selected and revealed no significant differences in functional outcomes between the two groups (RR = 1.02, 95%CI [0.81, 1.29], *P* = 0.84) (Fig. 9).

## Discussion

Our meta-analysis found no significant differences in fracture union time, union rate, union-related complications (malunion, nonunion, delayed union) of femoral neck and shaft fractures, osteonecrosis of the femoral head, and functional outcomes (Friedman-Wyman scoring system) (*P* > 0.05) during the treatment of ipsilateral femoral neck and shaft fractures with reconstruction nails or double implants.

Femoral neck fractures and femoral shaft fractures are prevalent during clinical practice [1], but ipsilateral fractures of both are rare [1, 2, 21]. The first documented case of this type of fracture was reported by Delaney et al. [45]. In 1958, the "dashboard femoral fracture" was proposed as the mechanism of injury for high-speed vehicle collisions, where the knee strikes the dashboard, creating an axial load on the femur with the knee and hip in flexed position, with the limb in neutral position or abducted [3, 22, 46]. It is now understood that shaft fractures are typically displaced [22] and comminuted, while neck fractures are usually vertical and nondisplaced, with an incidence rate of 25–60% [2, 4, 21].

Although various parameters were assessed in the present study, it was challenging to demonstrate the superiority of any particular approach in clinical outcomes. This meta-analysis revealed no significant difference in fracture union rates between the two fixation methods. Ostrum et al.'s multicenter study showed that double implants resulted in a 98% femoral neck fracture union rate and a 91.3% femoral shaft fracture union rate [30]. Khallaf et al. [33] found that all femoral neck fractures healed with double implants, while the femoral shaft healing rate was 76%. Jain et al. found that the healing rate of femoral neck and shaft fractures after reconstruction nails treatment was 95.2% and 81%, respectively [15]. That was to say, both fixation methods showed different degrees of healing rate after utilized.

Our study found no difference in the incidence of complications between reconstruction nails and dual implants. In contrast, a retrospective study conducted by Tsai et al. [7] found that the incidence of femoral neck fracture union complications in the treatment by dual implants was significantly higher than that in the reconstruction nails group. Given that using anterograde intramedullary nails led to a higher incidence of neck fracture union complications, the authors recommended against using anterograde intramedullary nails combined with cancellous screws for treating these fractures [7]. Bedi et al. [10] revealed that reconstruction nails caused fracture healing complications. In contrast, no fracture healing complications occurred in the double implants group, which led them to believe that reconstruction nails with high technical requirements were unsuitable for treating ipsilateral femoral neck and shaft fractures [10]. Similarly, Watson et al. [47] reported that complications of reconstruction nails were as high as 35%, and intramedullary nails combined with cancellous screws were associated with the least complications. Therefore, they did not recommend reconstruction nails but intramedullary nails combined with cancellous screws for the fractures. On the contrary, the study of Hossam et al. [48] suggested that reconstruction nails resulted in fewer healing-related complications and were suitable for treating ipsilateral femoral neck and shaft fractures. Kesemenli et al. [49] substantiated the superiority of intramedullary nails to screw-plate fixation in preventing nonunion and delayed union. These different results may be due to the experience and procedure of the surgeons.

Although there was no difference between the two groups in terms of osteonecrosis of the femoral head in our study, the incidence of osteonecrosis of the femoral head in the reconstruction nails group (5.2%) was higher than in the dual implants group (3.6%), consistent with reports that the incidence of osteonecrosis of the femoral head in ipsilateral femoral neck and shaft fractures ranges from 1.2% to 5% [50]. The incidence of reconstruction nails was higher than other fixation methods [50], which may be related to the operating techniques of surgeons.

The incidence of osteonecrosis of the femoral head of concomitant ipsilateral femoral neck and shaft fractures is lower than femoral neck fracture alone. In previous reports, the incidence of osteonecrosis of the femoral head in a single femoral neck fracture was 14.3% [51]. We attribute this outcome to the following factors. Firstly, in the present study, we found a higher incidence of Garden types I and II of ipsilateral femoral neck and shaft fractures compared to Garden types III and IV. It is now understood that most of the impact is absorbed by the femoral shaft, resulting in less energy transfer to the femoral neck [2]. Nondisplaced fractures fall under Garden types I and II, whereas displaced fractures come under Garden types III and IV [52]. Sundkvist et al. [53] found that the occurrence of displaced fractures was significantly higher compared to nondisplaced fractures in individual femoral neck fractures. It has been reported that nondisplaced fractures result in a lower rate of osteonecrosis of the femoral head compared to displaced fractures [54], corroborated by the meta-analysis conducted by Slobogean et al. [51]. Oh et al. [8] consistently believed that the only causative factor for femoral head osteonecrosis is a displaced femoral neck fracture. Secondly, Oh et al. [8] and Bedi et al. [10] suggested that the incidence of osteonecrosis could have been underestimated due to the short follow-up period. Therefore, a longer follow-up time may be required to accurately evaluate the risk of femoral head osteonecrosis.

Among the included studies, five studies [5, 7, 18, 19, 44] reported a longer operation time when using reconstruction nails compared to double implants due to the complexity of the former method. As SD was not reported, a meta-analysis could not be performed, leaving the significance of the difference unknown. Besides, two studies [7, 44] reported less intraoperative blood loss when using reconstruction nails than double implants, indicating a favorable feature of the former method. However, the data were insufficient to perform a meta-analysis, and statistical significance could not be inferred.

Although there is no difference between the two treatment methods. Given that reconstruction nails provide a closed reduction approach with biological fixation and offer benefits, including lesser trauma, reduced blood loss, and lower chances of postoperative infection, we suggest that this technique should be favored. If double implants are used, closed reduction internal fixation (retrograde intramedullary nails combined with cancellous screws) should be the preferred method wherever applicable.

Our study's limitations are that all studies included were retrospective studies and had a small sample size due to the low incidence of ipsilateral femoral neck and shaft fractures. Additionally, the high rate of missed diagnoses made it challenging to select the optimal treatment. These factors may have had some impact on the study's results. Thus, more large-sample, multicenter randomized controlled trials are warranted to increase the robustness of our findings.

## Conclusion

Based on our findings, we believe that reconstruction nails and dual implants both can treat ipsilateral femoral neck and shaft fractures, as both exhibit favorable surgical outcomes. The validity of this result is compromised by the type of the included studies, and the conclusion therefore should be interpreted with caution.

### Supplementary Information

**Additional file 1: Table S1. **Search strategy for PubMed. **Table S2.** Search strategy for Embase. **Table S3.** Search strategy for Cochrane Library. 

## Data Availability

The datasets supporting the conclusions of this article are included within the article and its additional files.

## Abbreviations

DHS/SHS: Dynamic/sliding hip screws
PRISMA: Preferred Reporting Items for Systematic Reviews and Meta-Analyses
RCTs: Randomized controlled trials
SD: Standard deviation
CI: Confidence interval
ADL: Activities of daily living
NOS: The Newcastle–Ottawa scale
SMD: Standardized mean difference
RR: Risk ratio
C: Control group
T: Treatment group
C/T: Treatment group /control group
M/F: Male/female
PC: Plate + cancellous screws
ANC: Antegrade nailing + cancellous screws
DHSP: Dynamic hip screws + plate
DHSR: Dynamic hip screws + retrograde femoral nailing
RC: Reconstruction nails
RNC: Retrograde nailing + cancellous screws
SHSR: Sliding hip screws + retrograde intramedullary nail
SHSN: Sliding hip screws + flexible nails

## References

[1] Peljovich AE, Patterson BM (1998). Ipsilateral femoral neck and shaft fractures. J Am Acad Orthop Surg.

[2] Hak DJ, Mauffrey C, Hake M, Hammerberg EM, Stahel PF (2015). Ipsilateral femoral neck and shaft fractures: current diagnostic and treatment strategies. Orthopedics.

[3] Marins MHT, Pallone LV, Vaz BAS, Ferreira AM, Nogueira-Barbosa MH, Salim R, et al. Ipsilateral femoral neck and shaft fractures. When do we need further image screening of the hip? Injury. 2021;52 Suppl 3:S65-s9. 10.1016/j.injury.2021.01.040. 10.1016/j.injury.2021.01.040 34083022

[4] Tornetta P, 3rd, Kain MS, Creevy WR. Diagnosis of femoral neck fractures in patients with a femoral shaft fracture. Improvement with a standard protocol. J Bone Joint Surg Am. 2007;89(1):39–43. 10.2106/jbjs.F.00297. 10.2106/JBJS.F.00297 17200308

[5] Singh R, Rohilla R, Magu NK, Siwach R, Kadian V, Sangwan SS (2008). Ipsilateral femoral neck and shaft fractures: a retrospective analysis of two treatment methods. J Orthop Traumatol.

[6] Cannada LK, Viehe T, Cates CA, Norris RJ, Zura RD, Dedmond B (2009). A retrospective review of high-energy femoral neck-shaft fractures. J Orthop Trauma.

[7] Tsai CH, Hsu HC, Fong YC, Lin CJ, Chen YH, Hsu CJ (2009). Treatment for ipsilateral fractures of femoral neck and shaft. Injury.

[8] Oh CW, Kim JW, Park KC, Apivatthakakul T, Luo CF, Wong MK (2022). Clinical outcomes and affecting factors of ipsilateral femoral neck and shaft fractures - Multination, multicenter analysis. J Orthop Sci.

[9] Angelini A, Mavrogenis AF, Crimì A, Georgoulis J, Sioutis S, Bekos A (2021). Double fractures of the femur: a review of 16 patients. Eur J Orthop Surg Traumatol.

[10] Bedi A, Karunakar MA, Caron T, Sanders RW, Haidukewych GJ (2009). Accuracy of reduction of ipsilateral femoral neck and shaft fractures–an analysis of various internal fixation strategies. J Orthop Trauma.

[11] Swiontkowski MF (1987). Ipsilateral femoral shaft and hip fractures. Orthop Clin North Am.

[12] Chen CH, Chen TB, Cheng YM, Chang JK, Lin SY, Hung SH (2000). Ipsilateral fractures of the femoral neck and shaft. Injury.

[13] Tsarouhas A, Hantes ME, Karachalios T, Bargiotas K, Malizos KN (2011). Reconstruction nailing for ipsilateral femoral neck and shaft fractures. Strategies Trauma Limb Reconstr.

[14] Alborno Y, Abunimer A, Abuodeh Y, Salameh M, Kayali H, Ahmed G (2022). The surgical outcomes of fixing ipsilateral femoral neck and shaft fractures: single versus double implants fixation. Eur J Orthop Surg Traumatol.

[15] Jain P, Maini L, Mishra P, Upadhyay A, Agarwal A (2004). Cephalomedullary interlocked nail for ipsilateral hip and femoral shaft fractures. Injury.

[16] Casey MJ, Chapman MW (1979). Ipsilateral concomitant fractures of the hip and femoral shaft. J Bone Joint Surg Am.

[17] Plancher KD, Donshik JD (1997). Femoral neck and ipsilateral neck and shaft fractures in the young adult. Orthop Clin North Am.

[18] Mohapatra N, Sethy G, Rana R. Ipsilateral fracture neck and shaft of femur: A prospective analysis of two methods. Journal of Orthopedics, Traumatology and Rehabilitation. 2017;9(1). 10.4103/jotr.jotr_16_16.

[19] K K. Ipsilateral femoral neck and shaft fractures: An analysis of two treatment methods. International Journal of Orthopaedics Sciences. 2017;3(2k):774–7. 10.22271/ortho.2017.v3.i3k.116.

[20] Swiontkowski MF, Hansen ST, Kellam J (1984). Ipsilateral fractures of the femoral neck and shaft. A treatment protocol. J Bone Joint Surg Am.

[21] Boulton CL, Pollak AN (2015). Special topic: Ipsilateral femoral neck and shaft fractures–does evidence give us the answer?. Injury.

[22] Jones CB, Walker JB (2018). Diagnosis and Management of Ipsilateral Femoral Neck and Shaft Fractures. J Am Acad Orthop Surg.

[23] Bose WJ, Corces A, Anderson LD (1992). A preliminary experience with the Russell-Taylor reconstruction nail for complex femoral fractures. J Trauma.

[24] Leung KS, So WS, Lam TP, Leung PC (1993). Treatment of ipsilateral femoral shaft fractures and hip fractures. Injury.

[25] Vidyadhara S, Rao SK (2009). Cephalomedullary nails in the management of ipsilateral neck and shaft fractures of the femur–one or two femoral neck screws?. Injury.

[26] Wang WY, Liu L, Wang GL, Fang Y, Yang TF (2010). Ipsilateral basicervical femoral neck and shaft fractures treated with long proximal femoral nail antirotation or various plate combinations: comparative study. J Orthop Sci.

[27] Garnavos C, Peterman A, Howard PW (1999). The treatment of difficult proximal femoral fractures with the Russell-Taylor reconstruction nail. Injury.

[28] Mohan K, Ellanti P, French H, Hogan N, McCarthy T (2019). Single versus separate implant fixation for concomitant ipsilateral femoral neck and shaft fractures: A systematic review. Orthop Rev (Pavia).

[29] Oh CW, Oh JK, Park BC, Jeon IH, Kyung HS, Kim SY (2006). Retrograde nailing with subsequent screw fixation for ipsilateral femoral shaft and neck fractures. Arch Orthop Trauma Surg.

[30] Ostrum RF, Tornetta P, Watson JT, Christiano A, Vafek E (2014). Ipsilateral proximal femur and shaft fractures treated with hip screws and a reamed retrograde intramedullary nail. Clin Orthop Relat Res.

[31] Ricci WM, Bellabarba C, Evanoff B, Herscovici D, DiPasquale T, Sanders R (2001). Retrograde versus antegrade nailing of femoral shaft fractures. J Orthop Trauma.

[32] Wei YP, Lin KC (2021). Dual-construct fixation is recommended in ipsilateral femoral neck fractures with infra-isthmus shaft fracture: A STROBE compliant study. Medicine (Baltimore).

[33] Khallaf F, Al-Mosalamy M, Al-Akkad M, Hantira H (2005). Surgical treatment for ipsilateral fractures of femoral neck and shaft. Med Princ Pract.

[34] Page MJ, McKenzie JE, Bossuyt PM, Boutron I, Hoffmann TC, Mulrow CD (2021). The PRISMA 2020 statement: an updated guideline for reporting systematic reviews. BMJ.

[35] Watanabe Y, Nishizawa Y, Takenaka N, Kobayashi M, Matsushita T (2009). Ability and limitation of radiographic assessment of fracture healing in rats. Clin Orthop Relat Res.

[36] Lim HS, Kim CK, Park YS, Moon YW, Lim SJ, Kim SM (2016). Factors Associated with Increased Healing Time in Complete Femoral Fractures After Long-Term Bisphosphonate Therapy. J Bone Joint Surg Am.

[37] Ng AJ, Yue B, Joseph S, Richardson M (2014). Delayed/non-union of upper limb fractures with bisphosphonates: systematic review and recommendations. ANZ J Surg.

[38] Fisher JS, Kazam JJ, Fufa D, Bartolotta RJ (2019). Radiologic evaluation of fracture healing. Skeletal Radiol.

[39] Hak DJ, Fitzpatrick D, Bishop JA, Marsh JL, Tilp S, Schnettler R (2014). Delayed union and nonunions: epidemiology, clinical issues, and financial aspects. Injury.

[40] Zalavras CG, Lieberman JR (2014). Osteonecrosis of the femoral head: evaluation and treatment. J Am Acad Orthop Surg.

[41] Friedman RJ, Wyman ET (1986). Ipsilateral hip and femoral shaft fractures. Clin Orthop Relat Res.

[42] Wells GA, Shea B, O’Connell D, Peterson J, Welch V, Losos M, et al. The Newcastle-Ottawa Scale (NOS) for assessing the quality of nonrandomised studies in meta-analyses Available from: http://www.ohri.ca/programs/clinical_epidemiology/oxford.htm

[43] Liu SJ, He W, Zhang DX, Fan YG (2008). Treatment for ipsilateral fractures of the femoral neck and shaft. Zhongguo Gu Shang.

[44] Feifan X, Junwu Y, Xihai Z, Jianhua G, Lian T, Yunkang Y (2020). Comparison of three different internal fixation methods in treatment of ipsilateral femoral neck and shaft fracture. J Tissue Eng.

[45] Delaney WM, Street DM (1953). Fracture of femoral shaft with fracture of neck of same femur; treatment with medullary nail for shaft and Knowles pins for neck. J Int Coll Surg.

[46] Ritchey SJ, Schonholtz GJ, Thompson MS (1958). The dashboard femoral fracture; pathomechanics, treatment, and prevention. J Bone Joint Surg Am.

[47] Watson JT, Moed BR (2002). Ipsilateral femoral neck and shaft fractures: complications and their treatment. Clin Orthop Relat Res.

[48] Hossam ElShafie M, Adel Morsey H, Emad EY (2001). Ipsilateral fracture of the femoral neck and shaft, treatment by reconstruction interlocking nail. Arch Orthop Trauma Surg.

[49] Kesemenli CC, Tosun B, Kim NS (2012). A comparison of intramedullary nailing and plate-screw fixation in the treatment for ipsilateral fracture of the hip and femoral shaft. Musculoskelet Surg.

[50] Bhandari M (2003). Ipsilateral femoral neck and shaft fractures. J Orthop Trauma.

[51] Slobogean GP, Sprague SA, Scott T, Bhandari M (2015). Complications following young femoral neck fractures. Injury.

[52] Kazley JM, Banerjee S, Abousayed MM, Rosenbaum AJ (2018). Classifications in Brief: Garden Classification of Femoral Neck Fractures. Clin Orthop Relat Res.

[53] Sundkvist J, Brüggeman A, Sayed-Noor A, Möller M, Wolf O, Mukka S (2021). Epidemiology, classification, treatment, and mortality of adult femoral neck and basicervical fractures: an observational study of 40,049 fractures from the Swedish Fracture Register. J Orthop Surg Res.

[54] Konarski W, Poboży T, Kotela A, Śliwczyński A, Kotela I, Hordowicz M, et al. The Risk of Avascular Necrosis Following the Stabilization of Femoral Neck Fractures: A Systematic Review and Meta-Analysis. Int J Environ Res Public Health. 2022;19(16). 10.3390/ijerph191610050. 10.3390/ijerph191610050 PMC9408780 36011686

