# Supplementary material for: Comparison of reconstruction nails versus dual implants in the treatment of ipsilateral femoral neck and shaft fractures in adults: a meta-analysis and systematic review
Source: BMC Musculoskelet Disord. 2023 Oct 9;24:800. doi: 10.1186/s12891-023-06933-6 (PMC10561477; doi:10.1186/s12891-023-06933-6)
Supplement: Supplementary file 1 — Additional file 1: Table S1. Search strategy for PubMed. Table S2. Search strategy for Embase. Table S3. Search strategy for Cochrane Library. [file 12891_2023_6933_MOESM1_ESM.docx]

**Table S1** Search strategy for PubMed

| Search number | Query | Results |
| --- | --- | --- |
| 25 | ((((("Femur Neck"[Mesh]) OR (((Neck, Femur) OR (Femoral Neck)) OR (Neck, Femoral))) AND (("Diaphyses"[Mesh]) OR ((Diaphysis) OR (shaft)))) AND (("Fractures, Bone"[Mesh]) OR (((((((((((((((((Broken Bones) OR (Bone, Broken)) OR (Bones, Broken)) OR (Broken Bone)) OR (Bone Fractures)) OR (Bone Fracture)) OR (Fracture, Bone)) OR (Spiral Fractures)) OR (Fracture, Spiral)) OR (Fractures, Spiral)) OR (Spiral Fracture)) OR (Torsion Fractures)) OR (Fracture, Torsion)) OR (Fractures, Torsion)) OR (Torsion Fracture)) OR (fracture)) OR (fractures)))) AND ((((("Bone Nails"[Mesh]) OR ((((((((((Bone Nail) OR (Nail, Bone)) OR (Nails, Bone)) OR (Bone Pins)) OR (Bone Pin)) OR (Pin, Bone)) OR (Pins, Bone)) OR (nail)) OR (nails)) OR (nailing))) OR (("Bone Screws"[Mesh]) OR (((((Bone Screw) OR (Screw, Bone)) OR (Screws, Bone)) OR (screw)) OR (screws)))) OR (("Bone Plates"[Mesh]) OR ((((((Bone Plate) OR (Plate, Bone)) OR (Plates, Bone)) OR (plate)) OR (plates)) OR (plating)))) OR (("Fracture Fixation"[Mesh]) OR (((((((((((((Fixation, Fracture) OR (Fixations, Fracture)) OR (Fracture Fixations)) OR (Skeletal Fixation)) OR (Fixation, Skeletal)) OR (Fixations, Skeletal)) OR (Skeletal Fixations)) OR (Fracture Reduction)) OR (Fracture Reductions)) OR (Reduction, Fracture)) OR (Reductions, Fracture)) OR (fixation)) OR (fixations))))) AND ((((reconstruction) OR (Cephalomedullary)) OR (retrograde)) OR (antegrade)) | 245 |
| 24 | (((("Bone Nails"[Mesh]) OR ((((((((((Bone Nail) OR (Nail, Bone)) OR (Nails, Bone)) OR (Bone Pins)) OR (Bone Pin)) OR (Pin, Bone)) OR (Pins, Bone)) OR (nail)) OR (nails)) OR (nailing))) OR (("Bone Screws"[Mesh]) OR (((((Bone Screw) OR (Screw, Bone)) OR (Screws, Bone)) OR (screw)) OR (screws)))) OR (("Bone Plates"[Mesh]) OR ((((((Bone Plate) OR (Plate, Bone)) OR (Plates, Bone)) OR (plate)) OR (plates)) OR (plating)))) OR (("Fracture Fixation"[Mesh]) OR (((((((((((((Fixation, Fracture) OR (Fixations, Fracture)) OR (Fracture Fixations)) OR (Skeletal Fixation)) OR (Fixation, Skeletal)) OR (Fixations, Skeletal)) OR (Skeletal Fixations)) OR (Fracture Reduction)) OR (Fracture Reductions)) OR (Reduction, Fracture)) OR (Reductions, Fracture)) OR (fixation)) OR (fixations))) | 495,574 |
| 23 | ((("Femur Neck"[Mesh]) OR (((Neck, Femur) OR (Femoral Neck)) OR (Neck, Femoral))) AND (("Diaphyses"[Mesh]) OR ((Diaphysis) OR (shaft)))) AND (("Fractures, Bone"[Mesh]) OR (((((((((((((((((Broken Bones) OR (Bone, Broken)) OR (Bones, Broken)) OR (Broken Bone)) OR (Bone Fractures)) OR (Bone Fracture)) OR (Fracture, Bone)) OR (Spiral Fractures)) OR (Fracture, Spiral)) OR (Fractures, Spiral)) OR (Spiral Fracture)) OR (Torsion Fractures)) OR (Fracture, Torsion)) OR (Fractures, Torsion)) OR (Torsion Fracture)) OR (fracture)) OR (fractures))) | 1,468 |
| 22 | ("Fracture Fixation"[Mesh]) OR (((((((((((((Fixation, Fracture) OR (Fixations, Fracture)) OR (Fracture Fixations)) OR (Skeletal Fixation)) OR (Fixation, Skeletal)) OR (Fixations, Skeletal)) OR (Skeletal Fixations)) OR (Fracture Reduction)) OR (Fracture Reductions)) OR (Reduction, Fracture)) OR (Reductions, Fracture)) OR (fixation)) OR (fixations)) | 262,481 |
| 21 | ("Bone Plates"[Mesh]) OR ((((((Bone Plate) OR (Plate, Bone)) OR (Plates, Bone)) OR (plate)) OR (plates)) OR (plating)) | 208,490 |
| 20 | ("Bone Screws"[Mesh]) OR (((((Bone Screw) OR (Screw, Bone)) OR (Screws, Bone)) OR (screw)) OR (screws)) | 58,371 |
| 19 | ("Bone Nails"[Mesh]) OR ((((((((((Bone Nail) OR (Nail, Bone)) OR (Nails, Bone)) OR (Bone Pins)) OR (Bone Pin)) OR (Pin, Bone)) OR (Pins, Bone)) OR (nail)) OR (nails)) OR (nailing)) | 55,557 |
| 18 | ("Fractures, Bone"[Mesh]) OR (((((((((((((((((Broken Bones) OR (Bone, Broken)) OR (Bones, Broken)) OR (Broken Bone)) OR (Bone Fractures)) OR (Bone Fracture)) OR (Fracture, Bone)) OR (Spiral Fractures)) OR (Fracture, Spiral)) OR (Fractures, Spiral)) OR (Spiral Fracture)) OR (Torsion Fractures)) OR (Fracture, Torsion)) OR (Fractures, Torsion)) OR (Torsion Fracture)) OR (fracture)) OR (fractures)) | 355,488 |
| 17 | ("Diaphyses"[Mesh]) OR ((Diaphysis) OR (shaft)) | 35,274 |
| 16 | ("Femur Neck"[Mesh]) OR (((Neck, Femur) OR (Femoral Neck)) OR (Neck, Femoral)) | 32,675 |
| 15 | (((reconstruction) OR (Cephalomedullary)) OR (retrograde)) OR (antegrade) | 663,046 |
| 14 | ((((((((((((Fixation, Fracture) OR (Fixations, Fracture)) OR (Fracture Fixations)) OR (Skeletal Fixation)) OR (Fixation, Skeletal)) OR (Fixations, Skeletal)) OR (Skeletal Fixations)) OR (Fracture Reduction)) OR (Fracture Reductions)) OR (Reduction, Fracture)) OR (Reductions, Fracture)) OR (fixation)) OR (fixations) | 262,481 |
| 13 | "Fracture Fixation"[Mesh] | 68,825 |
| 12 | (((((Bone Plate) OR (Plate, Bone)) OR (Plates, Bone)) OR (plate)) OR (plates)) OR (plating) | 208,490 |
| 11 | "Bone Plates"[Mesh] | 20,173 |
| 10 | ((((Bone Screw) OR (Screw, Bone)) OR (Screws, Bone)) OR (screw)) OR (screws) | 58,371 |
| 9 | "Bone Screws"[Mesh] | 28,130 |
| 8 | (((((((((Bone Nail) OR (Nail, Bone)) OR (Nails, Bone)) OR (Bone Pins)) OR (Bone Pin)) OR (Pin, Bone)) OR (Pins, Bone)) OR (nail)) OR (nails)) OR (nailing) | 55,557 |
| 7 | "Bone Nails"[Mesh] | 12,267 |
| 6 | ((((((((((((((((Broken Bones) OR (Bone, Broken)) OR (Bones, Broken)) OR (Broken Bone)) OR (Bone Fractures)) OR (Bone Fracture)) OR (Fracture, Bone)) OR (Spiral Fractures)) OR (Fracture, Spiral)) OR (Fractures, Spiral)) OR (Spiral Fracture)) OR (Torsion Fractures)) OR (Fracture, Torsion)) OR (Fractures, Torsion)) OR (Torsion Fracture)) OR (fracture)) OR (fractures) | 355,488 |
| 5 | "Fractures, Bone"[Mesh] | 203,802 |
| 4 | (Diaphysis) OR (shaft) | 35,274 |
| 3 | "Diaphyses"[Mesh] | 2,091 |
| 2 | ((Neck, Femur) OR (Femoral Neck)) OR (Neck, Femoral) | 32,675 |
| 1 | "Femur Neck"[Mesh] | 7,739 |

**Table S2** Search strategy for Embase

| No. | Query | Results |
| --- | --- | --- |
| #22 | #7 AND #20 AND #21 | 72 |
| #21 | 'reconstruction' OR 'cephalomedullary' OR 'retrograde' OR 'antegrade' | 513030 |
| #20 | #10 OR #13 OR #16 OR #19 | 585829 |
| #19 | #17 OR #18 | 285949 |
| #18 | 'bone fixation' OR 'bone fracture fixation' OR 'fixation, bone' OR 'fixation' OR 'fixations' | 253723 |
| #17 | 'fracture fixation'/exp | 104470 |
| #16 | #14 OR #15 | 259767 |
| #15 | 'bone fixation plate' OR 'bone plate, device' OR 'bone plates' OR 'fixation plate' OR 'fracture fixation plate' OR 'growth-correction orthopaedic fixation plate kit' OR 'growth-correction orthopedic fixation plate kit; ncb (device)' OR 'non bioabsorbable orthopedic fixation plate' OR 'non-bioabsorbable bone plate' OR 'non-bioabsorbable fixation plate' OR 'non-bioabsorbable orthopaedic fixation plate' OR 'non-sterile non-bioabsorbable orthopaedic fixation plate' OR 'non-sterile nonbioabsorbable orthopedic fixation plate' OR 'orthopaedic fixation plate, non-bioabsorbable, non-sterile' OR 'orthopaedic fixation plate, non-bioabsorbable, sterile' OR 'orthopaedic fixation plates' OR 'orthopedic fixation plate, non bioabsorbable' OR 'orthopedic fixation plate, nonbioabsorbable, non-sterile' OR 'orthopedic fixation plates' OR 'rival reduce' OR 'rival view' OR 'semitubular plate' OR 'sterile non-bioabsorbable orthopaedic fixation plate' OR 'tubular plate' OR 'plate' OR 'plates' OR 'plating' | 259767 |
| #14 | 'bone plate'/exp | 22926 |
| #13 | #11 OR #12 | 72366 |
| #12 | 'bone fixation nail' OR 'bone nails' OR 'multiloc phn' OR 'bone nail dummy' OR 'nail' OR 'nails' OR 'nailing' | 72353 |
| #11 | 'bone nail'/exp | 11624 |
| #10 | #8 OR #9 | 75058 |
| #9 | 'bone fixation screw' OR 'bone screws' OR 'fixation screw' OR 'intraosseous screw' OR 'leibinger (bone screw)' OR 'non bioabsorbable orthopedic bone screw' OR 'non-bioabsorbable orthopaedic bone screw' OR 'non-sterile non bioabsorbable orthopedic bone screw' OR 'non-sterile non-bioabsorbable orthopaedic bone screw' OR 'orthopaedic bone screw, non-bioabsorbable, non-sterile' OR 'orthopaedic bone screw, non-bioabsorbable, sterile' OR 'orthopedic bone screw, non bioabsorbable, non-sterile' OR 'orthopedic bone screw, non bioabsorbable, sterile' OR 'reimer screw' OR 'screw, fixation, bone' OR 'screws, bone' OR 'sterile non bioabsorbable orthopedic bone screw' OR 'sterile non-bioabsorbable orthopaedic bone screw' OR 'screw' OR 'screws' | 75001 |
| #8 | 'bone screw'/exp | 44805 |
| #7 | #3 AND #6 | 321 |
| #6 | #4 OR #5 | 3433 |
| #5 | 'femoral shaft fracture' OR 'fracture, femur shaft' | 1117 |
| #4 | 'femur shaft fracture'/exp | 3100 |
| #3 | #1 OR #2 | 14687 |
| #2 | 'femoral neck fractures' OR 'femur collum fracture' OR 'femur fracture, neck' OR 'femur neck fracture' OR 'fracture, femur neck' | 4821 |
| #1 | 'femoral neck fracture'/exp | 14132 |

**Table S3** Search strategy for Cochrane Library

| ID | Search | results |
| --- | --- | --- |
| #1 | MeSH descriptor: [Femur Neck] explode all trees | 489 |
| #2 | (Neck, Femoral OR Femoral Neck OR Neck, Femur) | 4692 |
| #3 | #1 OR #2 | 4692 |
| #4 | MeSH descriptor: [Diaphyses] explode all trees | 31 |
| #5 | (Diaphysis OR shaft) | 1691 |
| #6 | #4 OR #5 | 1700 |
| #7 | MeSH descriptor: [Fractures, Bone] explode all trees | 6958 |
| #8 | (Broken Bone OR Bone, Broken OR Broken Bones OR Bone Fractures OR Bones, Broken OR Bone Fracture OR Fracture, Bone OR Torsion Fracture OR Fractures, Torsion OR Spiral Fractures OR Fracture, Spiral OR Fractures, Spiral OR Spiral Fracture OR Torsion Fractures OR Fracture, Torsion OR fracture OR fractures) | 27548 |
| #9 | #7 OR #8 | 27577 |
| #10 | #3 AND #6 AND #9 | 148 |
| #11 | MeSH descriptor: [Bone Nails] explode all trees | 456 |
| #12 | (Nails, Bone OR Nail, Bone OR Bone Pin OR Pin, Bone OR Bone Pins OR Bone Nail OR Pins, Bone OR nail OR nails OR nailing) | 4184 |
| #13 | #11 OR #12 | 4184 |
| #14 | MeSH descriptor: [Bone Screws] explode all trees | 885 |
| #15 | (Bone Screw OR Screw, Bone OR Screws, Bone OR screw OR screws) | 4188 |
| #16 | #14 OR #15 | 4188 |
| #17 | MeSH descriptor: [Bone Plates] explode all trees | 682 |
| #18 | (Plate, Bone OR Plates, Bone OR Bone Plate OR plate OR plates OR plating) | 6695 |
| #19 | #17 OR #18 | 6695 |
| #20 | MeSH descriptor: [Fracture Fixation] explode all trees | 1976 |
| #21 | (Fixation, Skeletal OR Fracture Reductions OR Fracture Fixations OR Reduction, Fracture OR Reductions, Fracture OR Fixations, Fracture OR Fixation, Fracture OR Fixations, Skeletal OR Skeletal Fixations OR Skeletal Fixation OR Fracture Reduction OR fixation OR fixations) | 14042 |
| #22 | #20 OR #21 | 14048 |
| #23 | #13 OR #16 OR #19 OR #22 | 22780 |
| #24 | (reconstruction OR Cephalomedullary OR retrograde OR antegrade) | 14590 |
| #25 | #10 AND #23 AND #24 | 12 |
